# *Gtr9* mutation trades phage resistance for carbapenem sensitivity to potentiate phage-meropenem therapy against *carbapenem-resistant Acinetobacter baumannii in vitro*

**DOI:** 10.1128/aac.01355-25

**Published:** 2025-12-30

**Authors:** Jun Luo, Min Liu, Wen Ai, Xiaoling Zheng, Lu Han, Kuo Huang, Changlin Zhang, Jinhong Fan, Qianyuan Li, Chunhua Luo

**Affiliations:** 1 The First College of Clinical Medical Science, China Three Gorges University 26476, Yichang, China; 2 Yichang Central People's Hospital 159371, Yichang, China; University of Fribourg, Fribourg, Switzerland

**Keywords:** carbapenem-resistant *Acinetobacter baumannii*, phage-antibiotic synergy, carbapenem resistance attenuation, *Gtr9*, trade-off mechanism

## Abstract

The combined use of phages and antibiotics offers an alternative avenue against multidrug-resistant bacteria. We have previously described the synergistic antibacterial effect of the phage pB23 and meropenem combination against carbapenem-resistant *Acinetobacter baumannii* (CRAB). The study uncovers the underlying molecular mechanism of phage resistance in CRAB mediated by a novel stop-gain mutation in the gene *gtr9*. Through phenotypic characterization of pleiotropy, including reduction of capsular polysaccharide production and biofilm formation caused by the mutation in *gtr9*, we revealed an evolutionary trade-off mechanism whereby phage-resistant CRAB exhibits reduced carbapenem resistance. The zebrafish infection model demonstrated that these phage-resistant mutants were attenuated in virulence *in vivo*. Throughout continuous passage experiments *in vitro*, *gtr9* mutants displayed the stability of decreased growth rate, phage resistance, and virulence reduction. The combination therapy between phage pB23 and meropenem in different matrices exhibited consistent synergistic antibacterial activity *in vitro*, demonstrating its potential therapeutic *in vivo*. Collectively, our study reveals a trade-off mechanism underlying phage-antibiotic synergy, thereby providing a novel insight into bacterial resistance evolution and demonstrating the therapeutic potential of this approach against CRAB infections.

## INTRODUCTION

According to the WHO priority pathogens list published in 2024, carbapenem-resistant *Acinetobacter baumannii* (CRAB) was categorized as a critical priority pathogen (1). Invasive CRAB infections are associated with an increased risk of mortality by 8% to 40% (2), largely due to the lack of effective therapeutic options. For over 50 years, no new chemical-class antibiotics with activity against CRAB have been developed. Developing novel therapeutic strategies to combat infections caused by CRAB is urgently needed.

Phage therapy, an alternative to antibiotics in the age of multidrug resistance, has regained attention because of the escalating prevalence of drug-resistant bacteria and the slow development of novel antibiotics. Cases of phage-based therapy against drug-resistant *Acinetobacter baumannii* infections have been reported globally (3, 4). However, similar to monotherapy with antibiotics, bacteria also develop resistance to phages during phage monotherapy (5, 6). Consequently, the combination of phages and antibiotics has attracted increasing research interest due to its potential synergistic effects, which may reduce effective antibiotic concentrations and the emergence of phage-resistant and antibiotic-resistant bacteria. For instance, a new retrospective observational study illustrated that combined phage and antibiotic therapy significantly improved outcomes in difficult-to-treat infections caused by drug-resistant bacteria (such as *Pseudomonas aeruginosa* and *Staphylococcus aureus*), with 77.2% clinical improvement and 70% higher eradication rates versus phage alone (7). Similarly, a clinical cure for a recurrent, multidrug-resistant *Klebsiella pneumoniae* urinary tract infection was reported by leveraging phage-antibiotic synergy with an inactive drug (8).

We previously found that phage pB23 synergizes with inactive carbapenems against CRAB, although the mechanism remained unknown (9). To investigate this, we performed whole-genome sequencing of phage-resistant mutants and identified a stop-gain mutation in *gtr9*, a glycosyltransferase gene involved in capsular polysaccharide synthesis. Since the capsule is a critical receptor for phage adsorption (10), mutations in *gtr9* were likely to cause the phage-resistant phenotype. Phage resistance mediated by capsular alteration could produce a fitness trade-off, potentially affecting antibiotic sensitivity. A study demonstrated that combining colistin with a phage reduces resistance in *Acinetobacter baumannii* by altering the capsular structure (11). Similarly, another study showed that phage-resistant *Acinetobacter baumannii* strains exhibited increased susceptibility to ceftazidime due to impaired capsular synthesis (12). Furthermore, *gtr9* is also implicated in biofilm formation—a major resistance mechanism where bacterial communities exhibit up to 1000-fold increased tolerance to antimicrobials, largely due to impaired drug penetration and altered bacterial physiology (13).

**Fig 1:**
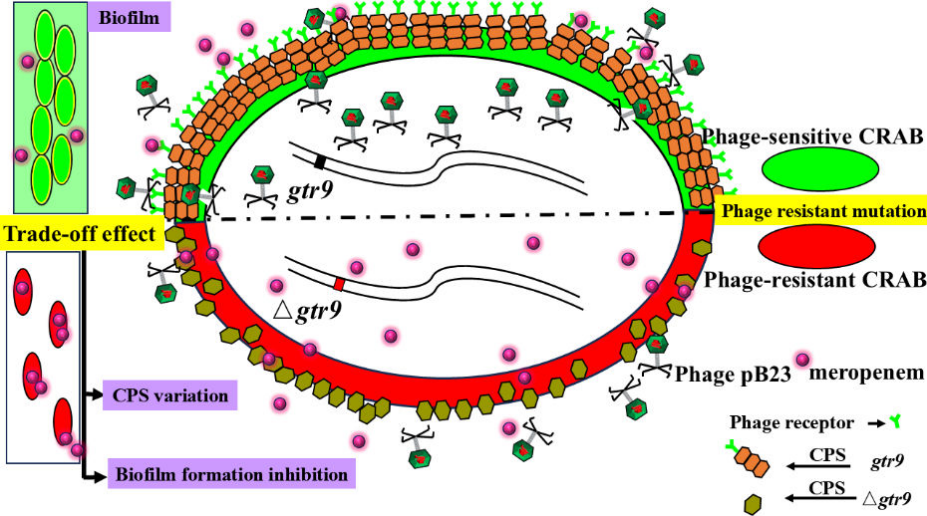
The trade-off effect of gene pleiotropy attenuates carbapenem resistance in phage-resistant CRAB mutants. Note: CPS (capsular polysaccharide). In phage-sensitive CRAB, the CPS serves as the receptor for phage pB23 adsorption. A stop-gain mutation in gene *gtr9* in CRAB not only confers phage resistance but also attenuates carbapenem resistance by pleiotropically reducing capsular polysaccharide production and biofilm formation.

Therefore, we propose the hypothesis that mutations in *gtr9* drive phage resistance, while their pleiotropic effects induce a trade-off effect that reduces carbapenem resistance in CRAB (illustrated in Fig. 1). This study aims to elucidate the underlying trade-off mechanism by which *gtr9* mutation confers phage resistance while simultaneously attenuating carbapenem resistance due to pleiotropic effects associated with reduction of capsular polysaccharide production and biofilm formation.

## MATERIALS AND METHODS

### Bacteria and phage solution preparation

*Acinetobacter baumannii* 2023 (B.m#2023) as the host bacteria, isolated from a sputum specimen in Yichang Central People’s Hospital (Hubei Province, China), was used in the present study. Bacterial suspension was prepared as previously reported (9). Briefly, strain, stored at −80°C with 25% glycerol, was routinely inoculated into Luria-Bertani agar plates (LB: 10 g of NaCl, 5 g of yeast extract, 10 g of tryptone, and 15 g of agar/L, where yeast extract, tryptone, and agar were purchased from Oxoid Ltd., Basingstoke, Hampshire, England) and incubated overnight at 37°C. Single colonies on agar plates were picked and inoculated into a shaking flask with 5 mL liquid LB medium. The shake flask was grown at 37°C and 180 rpm for 8 to 12 h. One milliliter of cultures was centrifuged at 6,000 rpm for 5 min. The bacterial pellet was resuspended in 1 mL LB broth after discarding the supernatant. Viable bacterial counts were based on the plate dilution method, and serial dilutions were prepared for the next experiments. According to CLSI standards (14), the antibiotic susceptibility profiles for B.m#2023 and mutants were determined using the broth microdilution method.

For the preparation of phage solution, phage enrichment was performed based on the soft agar double-layer technique. Five hundred microliters of host bacteria (B.m#2023, OD_600_ 0.8 to 1.2) and 100 μL of phage stored at −80°C with 25% glycerol were inoculated into LB liquid medium and grown overnight at 37°C with shaking at 180 rpm for 12 h. After incubation, the mixture was centrifuged at 12,000 rpm for 10 min, and the supernatant was filtered through a 0.22 μm membrane filter (Millipore Sigma, Bedford, MA). The phage titer was determined using the double agar overlay plaque assay as previously described (9).

### Isolation and identification of phage-resistant CRAB mutants

Phage-resistant CRAB mutants were isolated *in vitro* as described previously (9). Briefly, 100 µL of log-phase B.m#2023 culture was mixed with an equal volume of phage pB23 suspensions (MOI = 0.01) in a 96-well plate and incubated at 37°C. After 24 h, 10 µL of the mixture was streaked onto LB agar plates. Following overnight incubation at 37°C, a single colony was selected and cultured in LB broth for 12 h at 37°C with shaking at 180 rpm. The phage-resistant CRAB mutants were confirmed using two methods: the spot test and a phage amplification-TaqMan qPCR assay.

The spot test was performed as previously described (15). Briefly, 500 µL of overnight *B.m*#2023 culture were mixed with 5 mL of water agar and overlaid onto an LB agar plate. After solidification for 15 min, 5 µL of phage suspension (10⁸ PFU/mL) was spotted onto the agar surface. These plates were incubated at 37°C for 12 h, after which the presence or absence of a lytic zone was observed.

The phage amplification-TaqMan qPCR assay was performed by mixing equal volumes of bacteria and phage solutions (about 10^7^ CFU/mL and 10^7^ PFU/mL). Mixtures were incubated at 37°C and 180 rpm for 10 min, and the supernatant used as the template for qPCR was collected by centrifugation at 4°C for 5 min at 12,000 rpm. Primers and probes (Table 1), designed with Beacon Designer (v.8.13; Premier Biosoft International, USA), were synthesized by Sangon Biotech Co., Ltd. (Shanghai, China). The phage amplification-TaqMan qPCR assay was carried out in a 20 µL reaction system containing 10 µL of Premix Ex Taq (Probe qPCR; RR390A, Takara, China), 0.4 µL of each primer and probe (20 µM), 6.8 µL of ddH₂O, and 2 µL of template. DNase-free water served as the negative control. Amplification was performed under the following conditions: initial denaturation at 95°C for 3 min; 40 cycles of 95°C for 5 s and 60°C for 30 s, with fluorescence signal captured during the annealing step using a Quant Gene 9600 PCR system (Hangzhou Bioer Technology Co., Ltd., China).

**TABLE 1:** The sequences of the primers and the probe used in this study

Target		Sequence (5′–3′)	PCR product length
pB23	Forward	CAAGGATACTCTGGAGGA	121 bp
	Reverse	GGGATGTTAAAGTAAATGAGG	
	Probe	FAM- CCTAACACGGCAGCACGAGTA -BHQ1	

### Bacterial genome sequencing and analysis

The genomic DNA of the wild host bacteria B.m#2023 and the phage-resistant mutant were extracted using a Bacterial Genomic DNA Isolation Kit (Sangon Biotech, Shanghai, China) according to the manufacturer’s protocol. Purified DNA sequencing was performed by Sangon Biotech (Shanghai) Co., Ltd., China. Genomes were sequenced with Illumina HiSeq using the 150 bp paired-end approach. Raw reads were trimmed to eliminate low-quality reads and adapter sequences using Trimmomatic (version 0.39). Clean reads were aligned to the ATC1906 reference genome using GATK version 4.1.1.0. Mutations detected were subsequently manually reviewed using GATK version 4.1.1.0. Antibiotic resistance profile analysis of the wild host bacteria B.m#2023 was performed using ResFinder-4.7.2.

### Gene knockout and complementation experiment

Gene knockout of *gtr9* was performed based on the following description. The upstream and downstream homologous arms flanking the *gtr9* locus were amplified from wild B.m#2023 genomic DNA using a high-fidelity DNA polymerase. These arms were fused by overlap extension PCR to generate the knockout fragment *gtr9* (upstream arm-downstream arm), which was subsequently cloned into the suicide vector pCVD442AX (a pCVD442 derivative carrying an apramycin resistance gene), yielding the knockout plasmid pCVD442AX-*gtr9*. Then the plasmid was electroporated into *E. coli* β2155 to generate the donor strain β2155/pCVD442AX-*gtr9*. Conjugation between β2155/pCVD442AX-*gtr9* and wild-type *Acinetobacter baumannii* was performed. Apramycin-resistant *Acinetobacter baumannii* clones harboring single-crossover integrations (Aba/pCVD442AX-*gtr9*) were selected. Several *Acinetobacter baumannii*/pCVD442AX-*gtr9* clones were cultured overnight in antibiotic-free LB broth and plated on LB agar containing 10% sucrose. PCR and sequencing confirmed the successful generation of the *gtr9* T>A point mutant, designated *Acinetobacter baumannii gtr9* mutant (Δ*gtr9*). The phage adsorption ability of Δ*gtr9* was confirmed using two methods: the spot test and the phage amplification-TaqMan qPCR assay.

Gene complementation of Δ*gtr9* was also performed based on the following description. The apramycin-resistant plasmid pBBR1MCS2Apr (a pBBR1MCS2 derivative) was used as the complementation vector. The *gtr9* gene was amplified from wild B.m#2023 and cloned into pBBR1MCS2Apr via In-Fusion assembly at the EcoRV site, generating pBBR1MCS2Apr-*gtr9*. The plasmid was electroporated into *E. coli* WM3064 to produce the donor strain WM3064/pBBR1MCS2Apr-*gtr9*. Conjugation between WM3064/pBBR1MCS2Apr-*gtr9* and *Acinetobacter baumannii gtr9* mutant (Δ*gtr9*) was conducted. Apramycin-resistant transconjugants were selected, and PCR/sequencing verified the successful complementation. One clone was designated as the *gtr9*-complemented strain (Δ*gtr9::gtr9*). The phage adsorption ability of Δ*gtr9::gtr9* was confirmed using two methods: the spot test and the phage amplification-TaqMan qPCR assay.

### Fitness trade-off mediated by *gtr9* between phage resistance and carbapenem susceptibility

To further elucidate the mechanisms of carbapenem resistance attenuation in phage-resistant bacteria, we conducted the following investigations.

#### Assessing the growth kinetics of various bacteria

To compare the growth rates among the wild-type host bacteria B.m#2023 (W), phage-resistant bacteria (R), Δ*gtr9,* and Δ*gtr9::gtr9 in vitro*, overnight cultures were adjusted to 5 × 10⁷ CFU/mL. Then, 100 μL of bacterial suspension was inoculated into 12-well plates containing 1.9 mL of fresh LB broth per well. One hundred microliters of bacterial culture were collected at 0, 2, 4, 6 and 8 h, serially diluted, and plated for enumeration of viable bacteria. All experiments were performed in triplicate.

#### Biofilm formation assay

Overnight cultures of the wild-type host bacteria B.m#2023 (W), phage-resistant bacteria (R), Δ*gtr9,* and Δ*gtr9::gtr9* were diluted 1:100 (vol/vol) in fresh tryptic soy broth medium (Beijing Solarbio Science & Technology Co., Ltd.). Then, 400 μL aliquots were inoculated into 24-well plates. After 24 h incubation at 37°C, planktonic cells were removed by aspiration. Biofilms were gently washed three times with PBS and then resuspended in 1 mL PBS by scraping. The enumeration of viable bacteria was conducted by plating serial dilutions. All experiments were performed in triplicate.

#### Transmission electron microscopy

Five microliters of high-titer (10^9^ CFU/mL) wild-type host bacteria B.m#2023 (W), phage-resistant bacteria (R), Δ*gtr9* and Δ*gtr9::gtr9* suspensions were deposited on a carbon-coated copper grid and were allowed to adsorb for 1 min. Then, wild-type host bacteria B.m#2023 (W), phage-resistant bacteria (R), Δ*gtr9,* and Δ*gtr9::gtr9* particles were stained with 2% phosphotungstic acid (Beijing Solarbio Science & Technology Co., Ltd.). The carbon-coated copper grid was examined by transmission electron microscopy (TEM; JEM-F200; Jeol Co., Tokyo, Japan).

#### Capsular polysaccharide quantification

Uronic acids were quantified to assess capsule production according to the method of (16) with minor modifications. Briefly, 500 µL of overnight cultures of wild-type host bacteria B.m#2023 (W), phage-resistant bacteria (R), Δ*gtr9,* and Δ*gtr9::gtr9* (OD₆₀₀ = 1.0, about 2 × 10⁹ CFU/mL) was mixed with 100 µL of 1% zwittergent 3-14 in 100 mM citrate buffer (pH 2.0). After incubation at 50°C for 20 min, the mixture was centrifuged at 13,000 rpm for 5 min. The supernatant was collected, and capsular polysaccharides were precipitated overnight at 4°C using absolute ethanol. The precipitate was pelleted by centrifugation, air-dried, and resuspended in 200 µL ddH₂O. For hydrolysis, 1.2 mL of sodium tetraborate–sulfuric acid reagent (12.5 mM in H₂SO₄) was added, and the samples were boiled at 100°C for 5 min. After cooling on ice, 20 µL of 10% 3-hydroxydiphenol (in 0.5% NaOH) was introduced, and the mixture was shaken for 5 min. Absorbance was recorded at 520 nm using a microplate reader. Quantification was based on a uronic acid standard curve. The experiment was repeated in triplicate, and the error bars represent the standard deviations (SD).

### Virulence of *gtr9* mutants *in vivo*

To investigate the finding that the phage resistance-conferring gene influences bacterial pathogenicity *in vivo*, we assessed the survival rate of zebrafish infected with B.m#2023. Wild-type (AB) adult zebrafish mixed with males and females were housed with 14/10 h light/dark cycles at 28 ± 0.5°C. All protocols were performed in accordance with previously established procedures (9). Each fish in the control group (*n* = 12) was injected via the intraperitoneal (i.p.) route with 10 µL of PBS. Each fish in the experimental groups was injected via the i.p. route with 10 µL of the wild-type host bacteria B.m#2023 (W), phage-resistant bacteria (R), Δ*gtr9,* and Δ*gtr9::gtr9* at two concentrations (5 × 10^7^ and 5 × 10^4^ CFU/mL), respectively. The survival rate of zebrafish for each group at two concentrations was monitored via observation every 12 h until day 4 post-infection.

### Adaptive cost and long-term stability of the *gtr9* mutant *in vitro*

To further evaluate whether the fitness trade-off phenotype observed in phage-resistant bacteria remains stable over time, we performed a long-term serial passage experiment. Phage-resistant bacteria stored at −80°C were initially inoculated into a shaking flask with 5 mL liquid LB medium. The shake flask was grown for 12 h at 37°C with rolling at 180 rpm and the culture was designated passage zero (P0). To initiate a new passage, 50 µL of the current culture was transferred into a new shaking flask with 5 mL liquid LB medium. The new shake flask also was grown for 12 h at 37°C with rolling at 180 rpm. The cycle was repeated every 12 h, generating a total of 20 serial passages (P1 to P20). The growth rate, phage resistance, and virulence were assessed at designated passages (P0, P5, P10, P15, and P20). Bacterial growth rates were calculated according to the method reported previously with minor modification (17, 18). The growth rate constant (μ) was calculated using the exponential phase of the growth curve, with the formula μ = ln2/g, where g represents the doubling time. Phage resistance was evaluated using the spot test described above. Virulence was assessed by monitoring zebrafish survival over a 5 day period, according to the method in section Virulence of *gtr9* mutants *in vivo*.

### Synergistic antibacterial effects of phage and meropenem combination against CRAB in diverse matrices

Synergistic antibacterial effects of phage and meropenem combination in three different matrices including LB broth, bronchoalveolar lavage fluid, and serum were evaluated in 12-well plates according to the time-kill curve method. Prior to spiking, both bronchoalveolar lavage fluid and serum samples were tested by qPCR and plaque assays, which confirmed that neither B.m#2023 nor pB23 could be found. Bacterial and phage suspensions were adjusted to 4 × 10^7^ CFU/mL and 2 × 10^7^ PFU/mL, respectively. For the control group, 100 μL of bacterial suspension and 1.9 mL of matrix were added to wells. For the meropenem treatment group, 100 μL of bacterial suspension, 1.8 mL of matrix, and 100 µL of meropenem solution (20× MIC) were added to the wells. For the phage treatment group, 100 μL of bacterial suspension, 1.8 mL of matrix, and 100 µL of phage solution were added to wells. For the phage and meropenem combination treatment group, 100 μL of bacterial suspension, 1.7 mL of matrix, 100 µL of meropenem solution (20 × MIC), and 100 µL of phage solution were added to wells. These 12-well plates were incubated at 37°C, and viable bacterial counts were determined after 0, 4 h, 8 h, 24 h, 48 h, 72 h, 96 h, 120 h, and 144 h using the agar dilution plate-counting method. Data represent the mean values after performing the experiment in triplicate, and error bars represent the SD. Synergy was defined as a ≥2-log_10_-CFU/mL kill compared to the most effective agent after at least 6 h of incubation. Bactericidal activity is defined as a 3-log_10_-CFU/mL reduction from baseline. The experiment was repeated in triplicate.

### Statistical analysis

Mean values and standard deviations were computed with Microsoft Excel. For statistical analysis, GraphPad Prism (version 6.0; GraphPad Software Inc., USA) was employed. A two-sample *t*-test was applied to determine significance, with *P* < 0.05 considered statistically significant.

## RESULTS

### Identification of mutation conferring phage resistance

Whole-genome sequences of three phage-resistant mutants (R, R_1_, R_2_) and wild host bacteria B.m#2023 were mapped to the reference genome ATCC 19606 (GCA_009035845.1), respectively. Through comparative genomics and bioinformatic analysis, a stop-gain mutation (c.692T>A) in three mutants was identified in gene *gtr9*.

### Phage resistance mediated by a stop-gain mutation (c.692T>A) in *gtr9*

**Fig 2:**
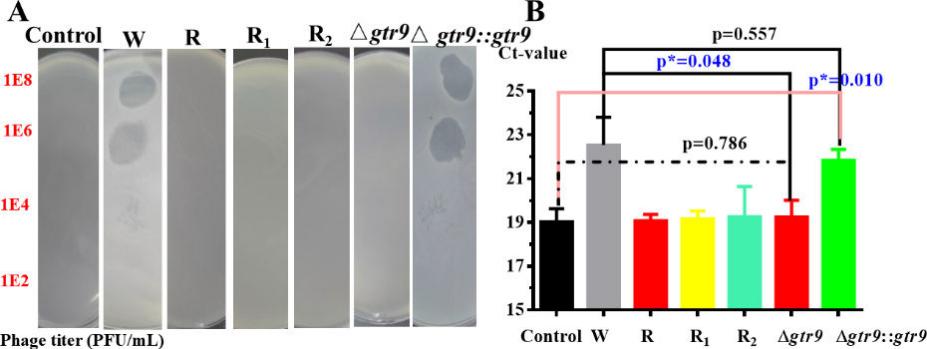
Identification of phage-resistant bacteria: the spot test (**A**) and phage amplification-TaqMan qPCR assay (**B**). Note: Control (phage pB23 alone). W (wild-type host bacteria B.m#2023). R/R_1_/R_2_(phage-resistant bacteria). Δ*gtr9* (gene knockout of *gtr9*). Δ*gtr9::gtr9* (*gtr9*-complemented strain). Representative results of the spot test showing the presence or absence of a lytic zone after dripping 5 µL of phage suspension (10⁸ PFU/mL) onto a lawn of B.m#2023 and incubating at 37°C for 12 h. The phage amplification-qPCR assay was performed by mixing equal volumes of bacterial and phage solutions (~10⁷ CFU/mL/PFU/mL), incubating at 37°C (180 rpm, 10 min), and collecting the supernatant via centrifugation (4°C, 12,000 rpm, 5 min) as the qPCR template. Error bars represent SD of three independent experiments (*, *P*-value <0.05).

No plaques were developed on the lawn of Δ*gtr9* (illustrated in Fig. 2A). While Δ*gtr9::gtr9* restored phage plaque formation to levels comparable to the wild host bacteria B.m#2023 (W). Quantitative analysis of phage adsorption (illustrated in Fig. 2B) demonstrated that the phage titer in Δ*gtr9* supernatant showed no significant difference compared to the control group (*P* = 0.786), but was significantly higher than that of the W (P* = 0.048 < 0.05). In contrast, the phage titer in Δ*gtr9::gtr9* supernatant was significantly reduced relative to the control (P* = 0.010 < 0.05), and no significant difference was observed compared to the W (*P* = 0.557 > 0.05). These results show that phage resistance in the mutant is caused by the stop-gain mutation (c.692T>A) in *gtr9*, which disrupts phage adsorption.

### Phenotypic characterization of phage-resistant mutants

#### Antibiotic resistance profiles

Antibiotic resistance profile analysis of the wild host bacteria B.m#2023 (W), performed by ResFinder-4.7.2, reveals that W harbors multiple resistance genes and is intrinsically resistant to a wide variety of antibiotics (shown in Table 2).

**TABLE 2:** Resistome prediction by ResFinder and antimicrobial susceptibility results acquired by the standard broth microdilution method^*a*^

Resistance genes of W		Antimicrobial susceptibility results of strains (μg/mL)				
Resistance gene	Spectrum of potential antibiotic resistance	Antibiotic	W	R	Δ*gtr9*	Δ*gtr9*::*gtr9*
*armA*	Amikacin, gentamicin, tobramycin, isepamicin, netilmicin	Amikacin	>512	>512	>512	>512
aph (6)-Id	Streptomycin	Gentamicin	>128	>128	>128	>128
aph(3'')-Ib	Streptomycin	Ciprofloxacin	>32	>32	>32	>32
aph(3')-Ia	Kanamycin, neomycin, lividomycin, paromomycin, ribostamycin	Cefotaxime	256	64	64	128
blaOXA-66	Beta-lactam	Meropenem	64	4	4	64
blaADC-25	Beta-lactam	Aztreonam	64	8	8	64
blaOXA-23	Imipenem, meropenem	Imipenem	>128	64	128	>128
blaTEM-1D	Amoxicillin, ampicillin, cephalothin, piperacillin, ticarcillin	Piperacillin	512	64	256	512
msr(E)	Erythromycin, azithromycin, quinupristin, pristinamycin IA, virginiamycin S	Tetracycline	>128	128	>128	>128
tet(B)	Doxycycline, tetracycline, minocycline	Minocycline	>128	16	32	>128

^*a*^ The antibiotic susceptibility profiles for strains were determined using the broth microdilution method. The breakpoints of the antibiotic panels were chosen according to the Clinical and Laboratory Standards Institute: amikacin (16 μg/mL), gentamicin (4 μg/mL), ciprofloxacin (1 μg/mL), cefotaxime (8 μg/mL)*, *meropenem (4 μg/mL), aztreonam (8 μg/mL)*, i*mipenem (4 μg/mL), piperacillin (16 μg/mL), tetracycline (4 μg/mL), minocycline (4 μg/mL). W (wild-type host bacteria B.m#2023). R (phage-resistant bacteria). Δ*gtr9 *(gene knockout of *gtr9*). Δ*gtr9*::*gtr9 *(*gtr9*-complemented strain).

Antibiotic susceptibility testing based on the broth microdilution method revealed a reduction in carbapenem resistance in the phage-resistant bacteria (R), which restored susceptibility to meropenem and aztreonam. The Δ*gtr9* exhibited a carbapenem resistance profile similar to that of R. In contrast, resistance in the Δ*gtr9::gtr9* was restored to the level of W, confirming that *gtr9* plays a significant role in carbapenem resistance.

#### Bacterial growth curve and biofilm formation analysis

**Fig 3:**
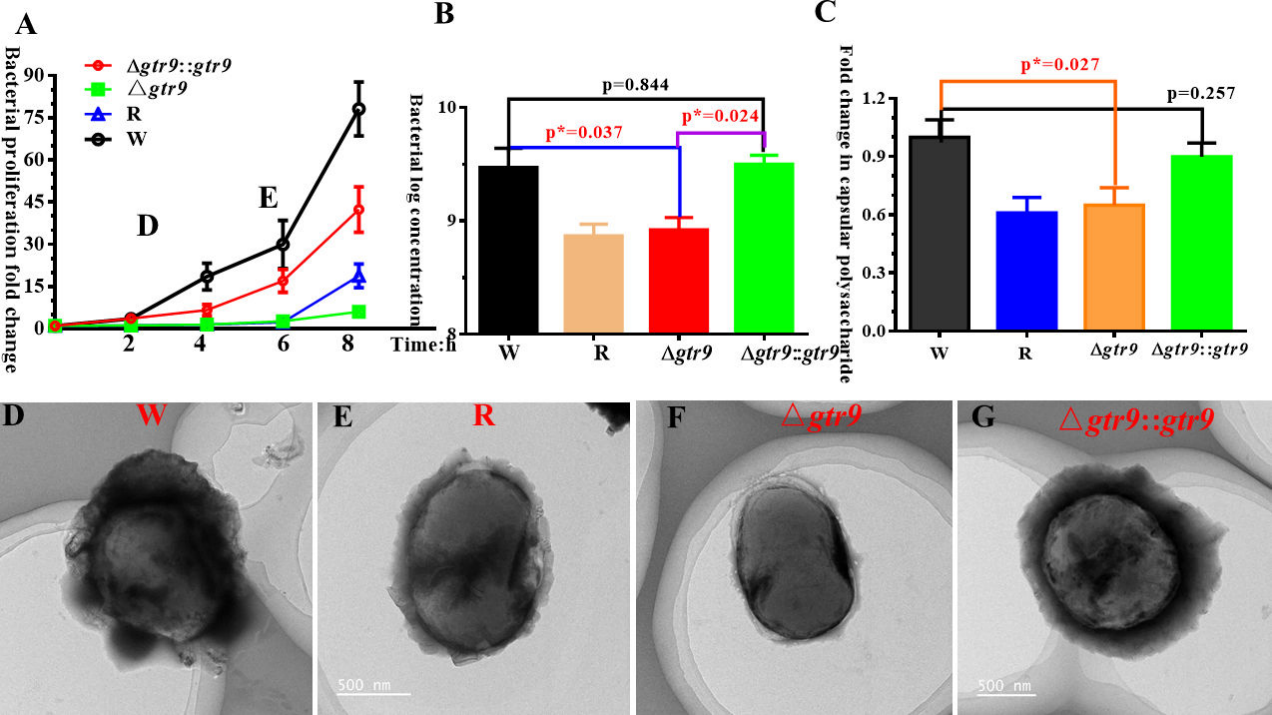
Phenotype analysis of W, R, Δ*gtr9,* and Δ*gtr9::gtr9*. (**A**) Bacterial growth curve. (**B**) Assessment of biofilm formation based on viable bacterial counts. (**C**) Capsule production was measured by uronic acid content, and normalization was performed using the W results as the reference. (**D–G**) Transmission electron micrograph. Note: W (wild-type host bacteria B.m#2023), R (phage-resistant bacteria), Δ*gtr9* (gene knockout of *gtr9*), Δ*gtr9::gtr9* (*gtr9*-complemented strain). Error bars represent SD of three independent experiments (*, *P*-value <0.05).

Δ*Gtr9* displayed obviously impaired growth kinetics, while Δ*gtr9::gtr9* restored growth rates to near those of the wild host bacteria B.m#2023 (W) levels (illustrated in Fig. 3A). Biofilm formation assay demonstrated that Δ*gtr9* exhibited a reduction in biofilm biomass compared to W (P* = 0.037). The Δ*gtr9::gtr9* recovered biofilm-forming capacity, showing no significant difference from W (*P* = 0.844) (illustrated in Fig. 3B).

#### Capsular polysaccharide quantification

Both the phage-resistant bacteria (R) and Δ*gtr9* exhibited a reduction in capsular polysaccharide (illustrated in Fig. 3C). Capsular polysaccharide production in Δ*gtr9* was significantly lower than that in the wild host bacteria B.m#2023 (W), with a statistically significant difference (P* = 0.027). In contrast, the Δ*gtr9::gtr9* restored capsular polysaccharide production to a level comparable to that of the W, with no significant difference (*P* = 0.257).

#### Morphological characterization of distinct bacterial strains

Distinct morphological differences were observed among these strains (illustrated in Fig. 3D through G). The wild host bacteria B.m#2023 (W) exhibited a predominantly coccoid morphology, surrounded by a prominent, hazy capsule layer that appeared significantly thicker than that of the phage-resistant bacteria (R)/Δ*gtr9*. In contrast, the R/Δ*gtr9* demonstrated an elongated cellular morphology with markedly reduced capsule formation around the cell periphery. The Δ*gtr9::gtr9* restored the W morphology, appearing coccoid and indistinguishable from W in capsule thickness.

### *Gtr9* mutants are less virulent *in vivo*

**Fig 4:**
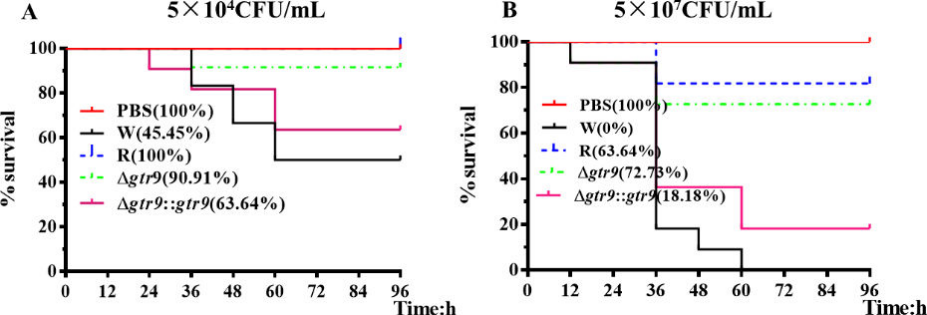
The survival curve of the zebrafish after inoculation with PBS buffer or different concentrations (**A**: 5 × 10^4^ and **B**: 5 × 10^7^ CFU/mL) of *Acinetobacter baumannii* solution. Note: W (wild-type host bacteria B.m#2023), R (phage-resistant bacteria), Δ*gtr9* (gene knockout of *gtr9*), Δ*gtr9::gtr9* (*gtr9*-complemented strain).

To investigate whether the phage resistance-conferring gene influences bacterial pathogenicity, we compared the virulence of various bacteria (W, R, Δ*gtr9,* and Δ*gtr9::gtr9*) using a zebrafish infection model. As shown in Fig. 4A and B, zebrafish infected with either the phage-resistant mutant or the Δ*gtr9* exhibited significantly higher survival rates than those infected with the W. In contrast, infection with the Δ*gtr9::gtr9* resulted in a notable reduction in survival compared to the Δ*gtr9*. These results indicate that mutation of *gtr9* markedly attenuates virulence, underscoring the critical role of *gtr9* in the pathogenicity of *A. baumannii*.

### Synergistic effects of phage and meropenem combination in different matrices

**Fig 5:**
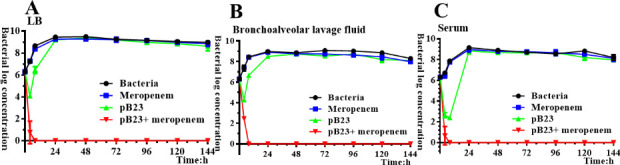
(**A–C**) Kinetic curve of combined use of phage pB23 and meropenem (4 µg/mL) in three different matrices. The *y*-axis represents the active bacterial amount, which is calculated as log (CFU/mL) using the dilution plate-counting method. The *x*-axis represents culture time.

After 4 h of incubation, both the phage alone and its combination with meropenem consistently demonstrated synergistic antibacterial effects across all three matrices (illustrated in Fig. 5 to Fig. 5C). However, extended incubation led to the emergence of phage-resistant populations in the phage monotherapy group. Notably, the combined phage-meropenem therapy retained significant bactericidal efficacy throughout a 144 h incubation period in all three matrices.

### Adaptive cost and long-term stability of the *gtr9* mutant under no phage/antibiotic pressure in continuous passage experiments *in vitro*

The phage-resistant bacteria at passage P0 exhibited a decreased growth rate compared to the wild host bacteria B.m#2023 (W), although the difference was not statistically significant (shown in Table 3). After 20 consecutive passages, the bacterial growth rate remained unchanged relative to P0, indicating no significant recovery or further fitness loss over time. Further analysis revealed that serial passages led to no change in virulence or in phage susceptibility.

**TABLE 3:** Adaptive cost and long-term stability of the *gtr9* mutant under no phage/antibiotic pressure continuous passage experiments *in vitro^a^*

Strain	Growth rate μ (h^−1^)	*P*	Zebrafish survival(%)	Phage susceptibility (S/R)
W	0.58 ± 0.09		25	S
P0	0.43 ± 0.02	*P*_0-W_ = 0.10	66.67	R
P5	0.37 ± 0.08	*P*_0-5_ = 0.396	75	R
P10	0.43 ± 0.12	*P*_0-10_ = 0.985	66.67	R
P15	0.35 ± 0.07	*P*_0-15_ = 0.374	58.33	R
P20	0.44 ± 0.06	*P*_0-20_ = 0.844	75	R

^*a*^ W (wild-type host bacteria B.m#2023), designated passages (P0, P5, P10, P15, and P20), *P *< 0.05 is considered statistically significant.

## DISCUSSION

The identification of *gtr9* through whole-genome sequencing of phage-resistant mutants reveals mutation in *gtr9* as a key genetic determinant of phage resistance in CRAB. While this aligns with known roles of glycosyltransferases in phage reception (19, 20), our study specifically demonstrates that a stop-gain mutation in *gtr9* confers this phenotype, which was confirmed by gene knockout and complementation. The associated reduction in capsular polysaccharide, quantified and visualized via TEM, directly links the mutation to a compromised phage adsorption site. Critically, this capsule deficiency presents a pleiotropic trade-off. Capsular polysaccharide thickness, a critical permeability barrier, is a key determinant of carbapenem resistance (21). Its attenuation by the *gtr9* mutation provides a mechanistic explanation for the observed reduction of carbapenem resistance, likely by enhancing antibiotic penetration and thus increasing the effective intracellular drug concentration. However, the precise structural alteration in the capsule that blocks phage adsorption remains unclear and needs future investigation.

Our finding that the *gtr9* mutation impairs biofilm formation provides a crucial mechanistic link to the observed carbapenem susceptibility. While biofilms are well-known to confer tolerance by acting as a physical barrier to antibiotic penetration (22, 23), the role of *gtr9* underscores that capsule synthesis is vital for constructing a robust biofilm matrix, as suggested previously (11). The convergence of both capsule reduction and biofilm deficiency in the *gtr9* mutant collectively undermines two major defensive layers of the bacterium, offering a compelling explanation for the pleiotropic attenuation of carbapenem resistance. Furthermore, reduced growth rate diminishes the activity of cell wall rebuilding and repair, thereby compromising its integrity and enhancing its permeability to cell wall-targeting antibiotics (24, 25).

The significantly attenuated virulence of phage-resistant mutants in the zebrafish model directly demonstrates the fitness cost associated with the *gtr9* mutation *in vivo*, supporting current reports that phage resistance often compromises pathogenicity (26, 27). However, we must acknowledge that relying solely on the zebrafish model is insufficient. Consequently, our findings must be validated in mammalian models. These systems provide critical physiological parallels to humans to better support the potential for clinical application of this strategy.

Continuous passage experiments of phage-resistant bacteria demonstrated that the attenuated virulence and other fitness costs incurred through evolutionary trade-offs remained stable over time. This stability, as corroborated by previous studies (28), may underlie the durable synergy of phage-antibiotic combinations. Furthermore, the combined application of phages and antibiotics across three distinct matrices revealed consistently potent and stable synergistic bactericidal effects. These cumulative findings collectively demonstrate the potential therapeutic synergy of this strategy *in vivo*.

In summary, this study reveals that a stop-gain mutation in the capsular polysaccharide gene *gtr9* confers phage resistance in CRAB at the cost of attenuated carbapenem resistance. This trade-off, driven by the pleiotropic reduction of both capsular polysaccharide and biofilm formation, highlights a vulnerable pathway in bacterial defense. By revealing a key gene for phage resistance, our study also provides a new avenue to combat CRAB by exploiting its evolutionary trade-off to weaken or reverse its drug resistance.

## Data Availability

The data that support the findings of this study are available on request from the corresponding author. The genome sequence of phage pB23 has been deposited to the GenBank database, and the accession number is OR994999.1.
